# PROTOCOL: The support needs of families living with parental mental illness: A qualitative systematic review

**DOI:** 10.1002/cl2.1267

**Published:** 2022-09-08

**Authors:** Emma Loudon, Gavin Davidson, Kathryn Higgins, Anne Grant

**Affiliations:** ^1^ Queen's Community and Place, School of Social Science, Education and Social Work Queen's University Belfast Belfast UK; ^2^ School of Social Sciences, Education & Social Work Queen's University Belfast Belfast UK; ^3^ School of Nursing and Midwifery Queen's University Belfast Belfast UK

## Abstract

This is the protocol for a Campbell systematic review. The objectives are as follows: To review existing qualitative research on the experiences of families living with parental mental illness from the perspective of (i) children, (ii) parents who have a mental illness) and (ii) the well parent. To synthesise qualitative evidence on the experience of living with parental mental illness and the experience of and attitude towards services from the perspective of (i) children, (ii) parents who have a mental illness and (ii) the well parent in order to develop the understanding of the needs of families and the implications for service provision.

## 1 BACKGROUND

### 1.1 The problem, condition or issue

Parental mental illness (PMI) is an issue that has received increasing attention in the academic literature over the past 20 years. The specific challenges and risks associated with mental illness (MI) in a parent have come to prominence in part due to child protection cases, where a child in the care of a parent with a mental health problem was serious harmed or killed (Social Care Institute for Excellence [SCIE], 2011; WHSSB & EHSSB, 2008). The outcomes of which included the lack of investigation of parenting status of mental health patients, failings in the interagency collaboration allowing for abuse to be detected, and insufficient engagement with children in families with PMI (Duffy et al., 2016; Falkov et al., 2016). The aims of research in this area vary by methodology and participant group but overall seek to understand how best to support families, either by examining how practitioners work (Grant et al., 2018; Luckock et al., 2017) testing interventions (Acri & Hoagwood, 2015; Foster et al., 2016) or gathering experiential data (Backer et al., 2017; Jones et al., 2016; Marston et al., 2018). This systematic review is interested in those studies which sought the lived experience of families, through qualitative methods; an area not always sufficiently prioritised by funding groups or policymakers. As the need to consult those with direct lived experience is increasingly recognised this area of research has grown, yet there continues to be gaps in the evidence base. Previous research in this area has frequently taken an individual approach focused on the unwell parent, and less often children, and with few exceptions, neglected the experience of other family members (Ahlström et al., 2009; Ballal et al., 2018; Price‐Robertson et al., 2015; SCIE, 2011). There has been even less research that has taken an integrative approach and sought the experience of the whole family affected by MI (Afzelius et al., 2018; Ahlström et al., 2009, Maybery et al., 2005) and yet families are crucial to understanding the experience of PMI, and for outcomes and recovery (Bland & Foster, 2012). The aim of the proposed review would be to evaluate the existing literature to provide evidence of the needs of families, to highlight any gaps and indicate future directions for research.

The prevalence of mental health problems in parents is difficult to estimate to exact figures due to underreporting and differences in recording and reporting measures. The mental health foundation attempts to estimate and report on prevalence, worldwide and in the United Kingdom (UK). For example, according to data collected within the last decade, worldwide, the rate of MI is thought to be around 10% of the population; this varies between countries with, for example, the UK at 14.5%, Ireland at 15.6% and the United States at 17.3% of the adult population (Mental Health Foundation, 2017). The most recent Adult Psychiatric Morbidity Survey was carried out in England and revealed that one in six people reported having a common mental health disorder at the time of the survey (i.e., depression or anxiety), and almost 50% of those surveyed reported symptoms of a mental health disorder at some point in their lifetime (McManus et al., 2016). Mental health problems can occur at any time over the life course, including while parenting, and in so doing impact the children of parents affected (Reupert & Maybery, 2016). Parenting can be a time of increased stress, and pregnancy and birth can be a trigger for mental health problems such as perinatal depression, anxiety and posttraumatic stress disorder (Howard et al., 2014).

Regarding the prevalence among parents, the Royal College of Psychiatrists report that that 68% of women and 57% of men with mental health problems are parents (2016). Furthermore, a national retrospective cohort study of UK children born between 1991 and 2015 provides perhaps the best estimates of children exposed to maternal mental illness (MMI) and indicates that overall, in the UK, the prevalence of MMI among these children was 23.2% (Abel et al., 2019). International figures provide a similar picture, with recent estimates suggesting that worldwide between 15% and 23% of children live with a parent with MI (Pierce et al., 2020). Similarly in a large‐scale study of young people and parents in Northern Ireland (NI) it was found that 22% of parents reported a previous diagnosis of a mental health disorder and that children of parents experiencing MI were twice as likely to themselves report symptoms such as anxiety or depression (Bunting et al., 2020). As can be seen there are different ways of reporting, however, the overall message of these figures is that many individuals are parenting with mental health issues, and many children are living in families where MI may impact their wellbeing and their outcomes.

It is well established in the literature that there are links between PMI and poor outcomes in children (Beardslee et al., 2012; Christiansen et al., 2015; Henshaw et al., 2011). The effects of PMI on children may be direct, such as in the case of predisposition to MI and behavioural problems in adolescence and adulthood (Melchior & van der Waerden, 2016). Indirect effects may include exposure to factors such as socioeconomic deprivation, relationship difficulties, domestic abuse, and co‐morbidities such as substance abuse (Fuller‐Thomson et al., 2019). In cases where children have died or been seriously injured, the investigations have found that PMI was a significant factor in the outcome (Duffy et al., 2016). While incidence of serious harm or death may be infrequent, children living with PMI may experience a range of adversities, which can lead to poorer educational attainment than their peers, behavioural and social difficulties, the strain of additional caring responsibilities and an increased risk of MI themselves (Davidson et al., 2012). However, the evidence suggests that there is not a linear causal relationship in the intergenerational transmission of MI (Landstedt & Almquist, 2019). There is evidence that around a third of children who grow up with a parent experiencing mental health difficulties do not experience negative outcomes in adulthood (Darlington et al., 2005). Not all families experiencing PMI will require intervention from services, and those that do are unlikely to universally conform to a particular set of needs or level of risk or respond to a specific intervention.

Despite the apparent public health concern created by PMI, too little research has been carried out that has explored the experience of the families affected and there have been calls for further research to be conducted especially with children (Cooklin, 2009; Reupert & Maybery, 2016). Some existing literature addressing children's perspectives explores the experience of young carers. While children may care for their parents due to a range of disabilities, there has been previous interest in those caring for a parent with MI (Aldridge, 2006; Aldridge & Becker, 2003; Cooklin, 2009; Obidina, 2010; SCIE, 2011). Aldridge (2006, p. 86) discussed the needs and experiences of children caring for a mentally ill parent and argued that while ‘children's development and childhood experience can be adversely affected when caring becomes long term and disproportionate’ young carers are not at inevitable risk of harm and that recognition of their role and contribution is highly valued.

As noted, there is a lack of research that deals with the partner or well parent experience and there is a significant gender imbalance with parenting often being conflated with mothering and a less research attending to MI in fathers (Fisher, 2016; Price‐Robertson et al., 2015). In general, there are fewer male voices in the qualitative research in this area either as the well or unwell parent although this is beginning to change. Recent research has begun to consider the perinatal mental health of fathers and the impact that this has on maternal mental health, infant mental health, and family wellbeing (Shorey & Chan, 2020). A small number of studies to date have taken a multiple‐perspective approach, by including the experience of parents and children and more rarely partner or whole families (Afzelius et al., 2018; Ahlström et al., 2009; Maybery et al., 2005). This type of approach is more inclusive, providing evidence from marginalised family members on the effects of PMI and additionally highlighting systemic effects not detectable in individual accounts. Similarly, a systematic review of the literature which incorporated a synthesis could help illustrate the experience of multiple members of the families affected, those previously given less attention and provide insight into the family as a unit. The synthesised data on multiple family members could provide additional support and evidence for those planning public services directed towards families with a mentally ill parent.

### 1.2 Description of the intervention

This qualitative systematic review will not be examining an intervention. The experiential data on the phenomena of interest becomes the outcome, in the absence of an intervention or experiment.

### 1.3 How the intervention might work

As above.

### 1.4 Why it is important to do this review

Despite the potential negative impact on outcomes for children there continues to be a lack of understanding of the experience of families, either in terms of gathering sufficient evidence from individual members or in providing integrated experiential accounts (SCIE, 2011). Internationally there has been a drive towards practice recommendations which support a whole family approach (Falkov, 1998, 2012; Goodyear et al., 2017; Reupert & Maybery, 2016; SCIE, 2011). Standards of practice that, at a minimum require adult mental health professionals to ask about parental status routinely, early intervention to promote family functioning, actively supporting children, working with parenting issues and the importance of interagency communication and collaboration (Davidson et al., 2012; Duffy et al., 2016). However, while countries all over the world are attempting to improve the approach to working with PMI, there is an acknowledged variability in the rate at which practice is changing (Falkov et al., 2016).

In July 2009, the Social Care Institute for Excellence published the ‘Think child, think parent, think family’ guide to help improve the response of services to parents with MI and their families in the UK (Diggns, 2011). The ‘Think family’ recommendations emphasised a ‘whole family’ approach underpinned by the Family Model, developed by Dr Adrian Falkov (1998, 2012). These recommendations have informed developments in family focused practice (FFP) within the UK for the last decade. Recently in NI, a large‐scale mixed methods study examined FFP from the perspective of professionals and parents who have MI (Grant et al., 2018). Findings suggested that while Think Family NI is a recognised initiative within some parts of the Health and Social Care system, levels of knowledge and understanding of FFP are variable. The report suggested that further research should be commissioned to ‘assist providers in better understanding how many families require help, what types of help are most effective for whom and in what circumstances, and to trial new interventions.’ (Grant et al., 2018). It is essential, of course, to conduct further primary studies with families; however, it is also important to synthesise the evidence which already exists to inform the direction of any planned research. To gain new understanding of the needs of families, a systematic review of the literature which incorporated a synthesis could provide unique insights and help illustrate the experience of multiple members of the families affected. The synthesised data on multiple family members could provide additional support and evidence for those planning public services directed towards families with a mentally ill parent.

As part of the study conducted by Grant et al. (2018), a systematic review was also conducted, which reviewed quantitative and qualitative studies of interventions with families experiencing MI or substance misuse in a parent. The research reviewed had to include interventions that were ‘family focused’, for example, that addressed the needs of at least a parent and child where a parent was experiencing MI or substance abuse. This review uncovered a range of interventions being used with families and reports of improvements because of FFP. The review provided some evidence for the perceived efficacy of FFP though no comparison with other interventions was made. In addition, due to the scope of this review it did not include qualitative research with parents exploring their perceptions of living with mental ill health or engaging with interventions, which might have provided further insight. Qualitative approaches, offer excellent ways to investigate families, allow a depth of understanding from an insider viewpoint and give voice to marginalised family members. It is nevertheless difficult to generalise from these studies due to the low levels of diversity in the sample and lack of representation of difficult to reach parents or those who have not accessed treatment. Consequently, public and social health organisations are making increasing use of qualitative systematic reviews to inform policy and practice, where data from multiple small‐scale studies can be synthesised increasing the strength of the evidence. (Gopalakrishnan & Ganeshkumar, 2013; Mills et al., 2005).

The proposed review would aim to provide a synthesis of existing qualitative research on PMI, which as has been noted is more frequently conducted with individual parents or children and rarely partners, rather than with family groups. Important insights have been gained from the existing research. For example, research with parents experiencing MI has uncovered some of the issues that this group are most concerned about, such as fears of losing their parenting responsibility, the importance their parenting role played in recovery and the need for professionals to acknowledge their parenting status (Jones et al., 2016; Marston et al., 2018; Van der Ende et al., 2016). Existing systematic reviews in this area include those that have synthesised the children's experiences as captured by qualitative research, for example, a meta‐synthesis by Dam and Hall (2016) which provided useful insight from 22 research studies with children of mentally ill parents. It highlighted the unpredictability of daily life faced by children living with a parent with MI and uncovered the shame and stigma children felt because of their parent's illness. It also suggested areas of focus for interventions such as the provision of information and education regarding PMI (Dam & Hall, 2016). These are important issues for policymakers and service providers to consider when assessing the needs and developing services for children. The proposed review would provide similarly useful information regarding not only children, but the whole family when there is PMI.

Reupert and Maybery (2016) provided an overview of the research field with a systematic review that took a multiple perspective approach and reviewed literature on the prevalence of MI in parents, the risk to children, the needs of children and parents and the experience of interventions. This review provided a comprehensive overview of the literature regarding families where a parent has a MI, from a range of viewpoints and in so doing uncovered a significant need for a ‘paradigm shift’ in the mental health services to meet families’ needs. Reupert and Maybery (2016) point out that their review was purposefully selective in that it sought to present a broad view of the research base illustrating the type and quality of research in this area. It provided not only an insight into the field but also uncovered the ways in which the problem is conceptualised, experienced and in some cases treated and at a level of complexity that a single perspective review would not have achieved. This proposed review would aim to discover and synthesise the qualitative data from families with experience of PMI to hopefully provide much needed information about their lived experiences, and the issues and problems that are most significant to this vulnerable group.

Taking a multiple perspective account of an issue by necessity increases the complexity of the data uncovered. In terms of the specific issue of PMI an approach that considers the whole family is one that finds agreement within some of the relevant theoretical perspectives such as family systems theory (FST). Based on the findings from previous research in this area, it is clear that multiple members of families where there is PMI experience adversity, including socioeconomic difficulties, decreased wellbeing and implications for children's welfare and family relationships and unity (Gladstone et al., 2006). FST understands the family to be an emotionally connected unit, likely to be disrupted by the mental health difficulties of an individual within the unit (Broderick, 1993). In FST it is emphasised that each part of the unit affects the functioning of the whole and that problems and issues exist within the context of the relationships (Becvar & Becvar, 2002). Family dynamics can have a significant impact on functioning and coping. Given the interconnectedness of the experiences within families it is significant to policy and practice that we gather as full a picture as possible which takes account of the nature, complexity and impact of these systems.

There have not been any literature reviews conducted that have sought to integrate and systematically review research regarding the experiences of all family members affected by PMI. Studies carried out with whole families have the potential to provide a complete picture of the impact and potential risks. Therefore, by employing a thematic meta‐synthesis of research which gathered the experience of parents, children, partners, or whole families the aim would be to generate new insights and understandings of the needs of families.

## 2 OBJECTIVES

The objectives of the review are:

To review existing qualitative research on the experiences of families living with PMI from the perspective of (i) children, (ii) parents who have a MI) and (ii) the well parent.

To synthesise qualitative evidence on the experience of living with PMI and the experience of and attitude towards services from the perspective of (i) children, (ii) parents who have a MI and (ii) the well parent to develop the understanding of the needs of families and the implications for service provision.

## 3 METHODS

### 3.1 Criteria for considering studies for this review

#### 3.1.1 Types of studies

It was established that SPIDER (Sample, Phenomenon of Interest, Design, Evaluation, Research type) was a more appropriate tool than the more commonly used PICO for developing the research question, search strategy and inclusion criteria. Therefore, for this evidence synthesis we will include qualitative studies that examined the experience of parents, partners and/or children (sample) living with PMI (phenomenon of interest). The following sections will discuss in more detail the design (primarily interview and focus group) and the reframing of ‘outcome data’ as the evaluation of experience and perception. In addition to purely qualitative research studies, we will also allow inclusion of qualitative results within mixed methods studies (research design). We will accept for inclusion studies from the last 20 years published in English. For further details regarding the methods used in the included studies see the section labelled ‘Description of methods used in primary research’.

#### 3.1.2 Types of participants

The sample (population of interest) in this review are parents both male and female who have experienced MI, their partners and children under the age of 18. Parents are defined as those who have parental responsibility for children through birth, marriage or adoption. It is expected that studies with child participants will include children who live at least part time with the unwell parent. Partners are defined as those who have lived with or supported a parent through close personal relationship while they experienced MI. For the purposes of this review those reviews which gathered the experience of children while they were in childhood (up to the age of 18) will be considered so that retrospective studies with adult children of mental ill parents will not be included. Other familial relationships will not be included, such as grandparents or sibling who might support due to PMI. There is little research in this area and the research that exists describes relationships that function in dynamically different ways to the parental family unit that is of interest here. The aim of this review is to discover the experience and needs of parents and children and comparison with the experience of extended family members would remove us from the specific field of enquiry.

#### 3.1.3 Types of interventions

The phenomenon of interest is the experience of families/family members living with PMI. Including but not necessarily limited to depression, anxiety, psychosis, schizophrenia, bipolar, personality disorder.

#### 3.1.4 Types of outcome measures

There is no need for an outcome statement in qualitative synthesis. The experiential data on the phenomena of interest becomes the outcome, in the absence of an intervention or experiment. The phenomena of interest in this study are the attitudes, beliefs, and experiences of parents who have suffered from mental ill health and their family members.

##### Types of settings

For the purposes of this review the context of the qualitative research may include community, in patient and outpatient settings.

### 3.2 Search methods for identification of studies

The strategy used was developed in consultation with a librarian and resulted in the search string; Mental illness OR Mental health OR Depression OR Anxiety OR Bipolar OR Schizophrenia AND Parental OR Parent OR Maternal OR Paternal OR Family OR COPMI AND Qualitative OR experience*.

#### 3.2.1 Electronic searches

Studies will be identified by searching the following databases.

1. Ovid MEDLINE
2. PsycINFO 2002‐ present
3. PubMed (MEDLINE)
4. CINAHL Complete
5. Cochrane Library
6. Social Sciences Citation Index (Web of Science)
7. Child Development & Adolescent Studies

#### 3.2.2 Searching other resources

Complementary searches will be used to address any deficits in the strategy including searching the reference list of included studies, google scholar and relevant websites to the topic.

### 3.3 Data collection and analysis

#### 3.3.1 Description of methods used in primary research

This review will consider studies from both interpretative and critical paradigms that focus on the collection and analysis of primary qualitative data. Methodologies including but not limited to, designs such as phenomenology, grounded theory, ethnography, and action research. Descriptive qualitative studies will also be considered. The data collection methods will include but are not limited to individual, family or joint interviews, focus groups and case studies. For the purposes of this review the context of the qualitative research may include community, inpatient and outpatient settings.

#### 3.3.2 Selection of studies

Two of the authors will independently screen titles and abstracts to exclude studies that are not relevant. Inclusion criteria is based on the SPIDER tool for qualitative evidence synthesis. Studies must be original qualitiative resesearch with members of families where a parent has a mental illness. Quantitative studies, systematic reviews and meta synthesises will be excluded. We will also exclude those reviews that focus on evaluating an intervention as we are concerned with sysnthesising experiential data. Studies considered eligible by at least one of the authors, or studies where there is insufficient information in the title and abstract to decide regarding inclusion will be retrieved in full text. Following this the full texts of studies will be read and assessed for inclusion independently by two of the review authors. Any disagreements on inclusion will be mediated by the third study author.

#### 3.3.3 Data extraction and management

Two review authors will independently extract data and enter this into NVivo for analysis. The data extracted will be reviewed by the third author. Qualitative findings may take the form of quotations from participants, subthemes and themes identified by authors. The Cochrane guidance also suggests that best practice involves extracting detailed contextual and methodological information on each study and reporting this information in a table of ‘characteristics of included studies’.

#### 3.3.4 Assessment of risk of bias in included studies

The CASP (Critical Skills Appraisal Programme) Qualitative checklist will be used to assess the risk of bias in all included studies. As advised by the Cochrane Handbook the studies will be assessed based on a ‘risk to rigour’ approach, that focuses on an overall assessment of methodological strength and weakness and the potential impact on findings. The CASP tool consists of ten question within three domains.

Are the results of the study valid?
What are the results?
Will the results help locally?

Within these domains are question designed to assess the methodological strengths and limitations. The CASP checklist will be applied to all included studies independently by two of the review authors. The CASP tool does not recommend a scoring system and the authors of this review found no disagreement with this. Due to the nature of qualitative data in this field and the interpretative aims of the synthesis the authors agreed that a CASP score was unnecessary. However, the authors propose that reports would be rejected based on the CASP assessment if they, for example, omitted important methodological details, lacked ethical considerations or if findings were poorly presented or absent. The results of the CASP screening of reports will be presented in table form. Following the quality assessment original qualitative data, comprising participant quotes and author interpretations, will be extracted for each included study, and entered into NVivo to enable meta‐synthesis. Data will be extracted by one reviewer and independently validated by a second reviewer.

#### 3.3.5 Assessment of reporting biases

See above.

#### 3.3.6 Data synthesis

The data that is extracted from the primary studies will be analysed using a thematic synthesis (Thomas & Harden, 2008). This process was chosen as it best fit the aims of the review to produce an interpretative synthesis through the development of analytic themes while descriptive themes maintain the link with the original research (2008). This process begins with findings in the form of quotes and themes from the primary studies are entered into the database and coded. The line‐by‐line coding leads to the development of firstly descriptive, then analytical themes. The development of descriptive themes requires the translation of concepts from one study to another, comparing and finding similarities and differences. The descriptive themes developed will then be used to develop analytical themes. The generation of analytical themes allows this method of synthesis to go beyond the primary studies interpretation of the data and occurs when the descriptive themes are used to uncover meanings and key messages from the data. The development of descriptive and then analytical themes will involve discussion between the review authors. Nvivo software will be used to support the thematic synthesis. It is hoped that individual perspectives can be retained within a whole family perspective as the themes incorporate the experience of multiple participants across a range of papers.

### 3.4 Sensitivity analysis

#### 3.4.1 Treatment of qualitative research

The details of the qualitative synthesis are above.

##### Summary of findings and assessment of the certainty of the evidence

This review plans to include a meta‐aggregative overview flowchart detailing the themes and synthesised findings. A data extraction table will also be included as would be standard in QES reporting. Further to this the findings of the evidence synthesis will be reported narratively theme by theme; studies relevant to that theme will be referenced using their numerical label from the data extraction table. This ensures that the findings of the thematic synthesis are supported by evidence from the included studies.

## CONTRIBUTIONS OF AUTHORS

- Content: Ms Emma Loudon, Dr Kathryn Higgins, Dr Gavin Davidson, Dr Anne Grant
- Systematic review methods: Ms Emma Loudon, Dr Kathryn, Higgins, Dr Gavin Davidson
- Statistical analysis: N/A
- Information retrieval: Ms Emma Loudon

## PRELIMINARY TIMEFRAME

Following approval of the protocol a 6‐month time frame for search, selection, data extraction and writing up of the systematic review is proposed resulting in a December 2020/January 2021 completion date.

## PLANS FOR UPDATING THIS REVIEW

Ms Emma Loudon has primary responsibility for updating the reviewing within the allocated timeframe.

## SOURCES OF SUPPORT

**Internal sources**

- No sources of support provided

**External sources**

- No sources of support provided

## References

[cl21267-bib-0002] Abel, K. M. , Hope, H. , Swift, E. , Parisi, R. , Ashcroft, D. M. , Kosidou, K. , Osam, C. S. , Dalman, C. , & Pierce, M. (2019). Prevalence of maternal mental illness among children and adolescents in the UK between 2005 and 2017: A national retrospective cohort analysis. The Lancet Public Health, 4(6), e291–e300. 31155222 10.1016/S2468-2667(19)30059-3 PMC6557735

[cl21267-bib-0003] Acri, M. C. , & Hoagwood, K. E. (2015). Addressing parental mental health within interventions for children: A review. Research on Social Work Practice, 25(5), 578–586. 26527857 10.1177/1049731514546027 PMC4627715

[cl21267-bib-0004] Afzelius, M. , Plantin, L. , & Östman, M. (2018). Families living with parental mental illness and their experiences of family interventions. Journal of Psychiatric and Mental Health Nursing, 25(2), 69–77. 28906576 10.1111/jpm.12433

[cl21267-bib-0005] Ahlström, B. H. , Skärsäter, I. , & Danielson, E. (2009). Living with major depression: experiences from families’ perspectives. Scandinavian Journal of Caring Sciences, 23(2), 309–316. 19302376 10.1111/j.1471-6712.2008.00624.x

[cl21267-bib-0006] Aldridge, J. (2006). The experiences of children living with and caring for parents with mental illness. Child Abuse Review, 15(2), 79–88.

[cl21267-bib-0007] Aldridge, J. , & Becker, S. (2003). Children caring for parents with mental illness: Perspectives of young carers, parents and professionals. The Policy Press.

[cl21267-bib-0008] Backer, C. , Murphy, R. , Fox, J. R. , Ulph, F. , & Calam, R. (2017). Young children's experiences of living with a parent with bipolar disorder: Understanding the child's perspective. Psychology and Psychotherapy: Theory, Research and Practice, 90(2), 212–228. 10.1111/papt.12099 27432719

[cl21267-bib-0053] Ballal, D ., & Navaneetham, J . (2018). Talking to children about parental mental illness: The experiences of well parents. International Journal of Social Psychiatry, 64(4), 367–373. 10.1177/0020764018763687 29536794

[cl21267-bib-0009] Beardslee, W. R. , Solantaus, T. S. , Morgan, B. S. , Gladstone, T. R. , & Kowalenko, N. M. (2012). Preventive interventions for children of parents with depression: International perspectives. Medical Journal of Australia, 196(7), 23–25. 10.5694/mja11.11289 25369844

[cl21267-bib-0010] Becvar, D. , & Becvar, R. (2002). Family therapy: A systemic integration. Pearson Education Australia.

[cl21267-bib-0054] Bland, R ., & Foster, M . (2012). Families and mental illness: Contested perspectives and implications for practice and policy. Australian Social Work, 65(4), 517–534. 10.1080/0312407X.2011.646281

[cl21267-bib-0011] Broderick, C. B. (1993). Understanding family process: Basics of family systems theory. Sage.

[cl21267-bib-0012] Bunting, L. , McCartan, C. , Davidson, G. , Grant, A. , McBride, O. , Mulholland, C. , Murphy, J. , Schubotz, D. , Cameron, J. , & Shevlin, M. (2020). *The mental health of children and parents in Northern Ireland: Results of the youth wellbeing prevalence survey*. Final Report.

[cl21267-bib-0013] Christiansen, H. , Anding, J. , Schrott, B. , & Röhrle, B. (2015). Children of mentally ill parents‐a pilot study of a group intervention program. Frontiers in Psychology, 6, 1494. 26539129 10.3389/fpsyg.2015.01494 PMC4611090

[cl21267-bib-0014] Cooklin, A. (2009). Children as carers of parents with mental illness. Psychiatry, 8(1), 17–20.

[cl21267-bib-0015] Dam, K. , & Hall, E. O. (2016). Navigating in an unpredictable daily life: A metasynthesis on children's experiences living with a parent with severe mental illness. Scandinavian Journal of Caring Sciences, 30(3), 442–457. 26763757 10.1111/scs.12285

[cl21267-bib-0016] Darlington, Y. , Feeney, J. A. , & Rixon, K. (2005). Interagency collaboration between child protection and mental health services: Practices, attitudes and barriers. Child Abuse & Neglect, 29(10), 1085–1098. 16315352 10.1016/j.chiabu.2005.04.005

[cl21267-bib-0017] Davidson, G. , Duffy, J. , Barry, L. , Curry, P. , Darragh, E. , & Lees, J. (2012). Championing the interface between mental health and child protection: Evaluation of a service initiative to improve joint working in Northern Ireland. Child Abuse Review, 21(3), 157–172.

[cl21267-bib-0018] Diggns, M. (2011). Think child, think parent, think family: A guide to parental mental health and child welfare. Social Care Institute for Excellence.

[cl21267-bib-0019] Duffy, J. , Davidson, G. , & Kavanagh, D. (2016). Applying the recovery approach to the interface between mental health and child protection services. Child Care in Practice, 22(1), 35–49.

[cl21267-bib-0020] Falkov, A. (1998). Crossing bridges: Training resources for working with mentally ill parents and their children, reader‐for managers, practioners and trainers. Department of Health.

[cl21267-bib-0021] Falkov, A. (2012). The family model handbook: An integrated approach to supporting mentally ill parents and their children. Pavilion.

[cl21267-bib-0022] Falkov, A. , Goodyear, M. , Hosman, C. M. , Biebel, K. , Skogøy, B. E. , Kowalenko, N. , Wolf, T. , & Re, E. (2016). A systems approach to enhance global efforts to implement family‐focused mental health interventions. Child & Youth Services, 37(2), 175–193.

[cl21267-bib-0023] Fisher, S. D. (2016). Paternal mental health: Why is it relevant? American Journal of Lifestyle Medicine, 11(3), 200–211. 30202331 10.1177/1559827616629895 PMC6125083

[cl21267-bib-0024] Foster, K. , Maybery, D. , Reupert, A. , Gladstone, B. , Grant, A. , Ruud, T. , Falkov, A. , & Kowalenko, N. (2016). Family‐focused practice in mental health care: An integrative review. Child & Youth Services, 37(2), 129–155.

[cl21267-bib-0025] Fuller‐Thomson, E. , Sawyer, J. L. , & Agbeyaka, S. (2019). The toxic triad: Childhood exposure to parental domestic violence, parental addictions, and parental mental illness as factors associated with childhood physical abuse. Journal of Interpersonal Violence, 36, 9015. 10.1177/0886260519853407 31184531

[cl21267-bib-0026] Gladstone, B. M. , Boydell, K. M. , & McKeever, P. (2006). Recasting research into children's experiences of parental mental illness: Beyond risk and resilience. Social Science & Medicine, 62(10), 2540–2550. 16316714 10.1016/j.socscimed.2005.10.038

[cl21267-bib-0027] Goodyear, M. , Maybery, D. , Reupert, A. , Allchin, R. , Fraser, C. , Fernbacher, S. , & Cuff, R. (2017). Thinking families: A study of the characteristics of the workforce that delivers family‐focussed practice. International Journal of Mental Health Nursing, 26(3), 238–248. 28026142 10.1111/inm.12293

[cl21267-bib-0028] Gopalakrishnan, S. , & Ganeshkumar, P. (2013). Systematic reviews and meta‐analysis: understanding the best evidence in primary healthcare. Journal of Family Medicine and Primary Care, 2(1), 9–14. 10.4103/2249-4863.109934 PMC3894019 24479036

[cl21267-bib-0029] Grant, A. , Lagdon, S. , Devaney, J. , Davidson, G. , Duffy, J. , Perra, O. , Galway, K. , Leavey, G. , & Monds‐Watson, A. (2018). A study of health and social care professionals’ family focused practice with parents who have mental illness, their children and families in Northern Ireland. Final Report. Queen's University Belfast.

[cl21267-bib-0030] Henshaw, C. , Adshead, G. , & Bende, B. (2011). *Parents as patients: supporting the needs of patients who are parents and their children* (Royal College of Psychiatrists Report CR164).

[cl21267-bib-0031] Howard, L. M. , Piot, P. , & Stein, A. (2014). No health without perinatal mental health. The Lancet, 384, 1723–1724. 10.1016/S0140-6736(14)62040-7 25455235

[cl21267-bib-0032] Jones, M. , Pietilä, I. , Joronen, K. , Simpson, W. , Gray, S. , & Kaunonen, M. (2016). Parents with mental illness—A qualitative study of identities and experiences with support services. Journal of Psychiatric and Mental Health Nursing, 23(8), 471–478. 27500507 10.1111/jpm.12321

[cl21267-bib-0033] Landstedt, E. , & Almquist, Y. B. (2019). Intergenerational patterns of mental health problems: The role of childhood peer status position. BMC Psychiatry, 19(1), 1–10. 31533680 10.1186/s12888-019-2278-1 PMC6749655

[cl21267-bib-0034] Luckock, B. , Barlow, J. , & Brown, C. (2017). Developing innovative models of practice at the interface between the NHS and child and family social work where children living at home are at risk of abuse and neglect: A scoping review. Child & Family Social Work, 22(s4), 62–69.

[cl21267-bib-0035] Manning, C. , & Gregoire, A. (2006). Effects of parental mental illness on children. Psychiatry, 5(1), 10–12.

[cl21267-bib-0036] Marston, N. , Maybery, D. , & Reupert, A. (2018). Parent perceptions comparing times of parental mental wellness and illness using a family functioning model. Journal of Family Studies, 24(3), 235–256.

[cl21267-bib-0037] Maybery, D. , Ling, L. , Szakacs, E. , & Reupert, A. (2005). Children of a parent with a mental illness: Perspectives on need. Australian e‐Journal for the Advancement of Mental Health, 4(2), 78–88.

[cl21267-bib-0038] McManus, S. , Bebbington, P. , Jenkins, R. & Brugha, T. (Eds.). (2016). Mental health and wellbeing in England: Adult Psychiatric Morbidity Survey 2014. NHS Digital.

[cl21267-bib-0039] Melchior, M. , & van der Waerden, J. (2016). Parental influences on children's mental health: The bad and the good sides of it. European Child & Adolescent Psychiatry, 25(8), 805–807. 27456961 10.1007/s00787-016-0891-9

[cl21267-bib-0040] Mental Health Foundation . (2017). *Mental health statistics*. mentalhealth.org

[cl21267-bib-0041] Mills, E. , Jadad, A. R. , Ross, C. , & Wilson, K. (2005). Systematic review of qualitative studies exploring parental beliefs and attitudes toward childhood vaccination identifies common barriers to vaccination. Journal of Clinical Epidemiology, 58(11), 1081–1088. 16223649 10.1016/j.jclinepi.2005.09.002

[cl21267-bib-0042] Obidina . (2010). Parental mental illness: Effects on young carers. British Journal of School Nursing, 5(3), 135–139.

[cl21267-bib-0043] Pierce, M. , Abel, K. M. , Muwonge, J., Jr. , Wicks, S. , Nevriana, A. , Hope, H. , Dalman, C. , & Kosidou, K. (2020). Prevalence of parental mental illness and association with socioeconomic adversity among children in Sweden between 2006 and 2016: A population‐based cohort study. The Lancet Public Health, 5(11), e583–e591. 33120044 10.1016/S2468-2667(20)30202-4

[cl21267-bib-0044] Price‐Robertson, R. , Reupert, A. , & Maybery, D. (2015). Fathers’ experiences of mental illness stigma: scoping review and implications for prevention. Advances in Mental Health, 13(2), 100–112.

[cl21267-bib-0045] Reupert, A. , & Maybery, D. (2016). What do we know about families where parents have a mental illness? A systematic review. Child & Youth Services, 37(2), 98–111.

[cl21267-bib-0046] Royal College of Psychiatrists . (2011). Parents as patients: Supporting the needs of parents who are patients and their children. College report.

[cl21267-bib-0047] Social Care Institute for Excellence (SCIE) . (2011). Think child, think parent, think family: A guide to parental mental health and child welfare.

[cl21267-bib-0048] Shorey, S. , & Chan, V. (2020). Paternal mental health during the perinatal period: A qualitative systematic review. Journal of Advanced Nursing, 76(6), 1307–1319. 32043615 10.1111/jan.14325

[cl21267-bib-0049] Thomas, J. , & Harden, A. (2008). Methods for the thematic synthesis of qualitative research in systematic reviews. BMC Medical Research Methodology, 8(1), 45. 18616818 10.1186/1471-2288-8-45 PMC2478656

[cl21267-bib-0050] Van der Ende, P. C. , Van Busschbach, J. T. , Nicholson, J. , Korevaar, E. L. , & Van Weeghel, J. (2016). Strategies for parenting by mothers and fathers with a mental illness. Journal of Psychiatric and Mental Health Nursing, 23(2), 86–97. 26868044 10.1111/jpm.12283

[cl21267-bib-0051] WHSSB & EHSSB . (2008). Report of the independent inquiry panel to the Western & Eastern Health and Social Services Boards: Madeleine and Lauren O'Neill . Government Report.

